# A Novel Variant of the LMAN1 Gene in Combined Factor V and Factor VIII Deficiency in a Saudi Female Child: A Case Report

**DOI:** 10.7759/cureus.56858

**Published:** 2024-03-24

**Authors:** Badriah G Alasmari, Mohammed Alpakra, Syed Rayees, Sara S Hassanien, Zainab E Almakki

**Affiliations:** 1 Department of Pediatrics, Armed Forces Hospital Southern Region, Khamis Mushait, SAU; 2 Department of Hematology and Oncology, Armed Forces Hospital Southern Region, Khamis Mushait, SAU; 3 Department of Hematology, Maternity and Children Hospital (MCH), Dammam, SAU

**Keywords:** pediatric, genetics, hematology, combined factor deficiency, lman1, factor viii, factor v

## Abstract

Combined factor V and factor VIII deficiency (F5F8D) is an exceedingly rare autosomal recessive disease that causes concomitantly low levels of factor V and factor VIII, leading to mild to moderate bleeding tendencies. Within this disorder, mutations manifest in the lectin mannose-binding protein (LMAN1) or multiple coagulation factor deficiency 2 (MCFD2) genes. This report presents a case of a five-year-old Saudi female child who was referred from an otolaryngology clinic, with an incidental finding of prolonged prothrombin time (PT), international normalized ratio (INR), and activated partial thromboplastin time (aPTT) detected during routine preoperative investigations for tonsillectomy, prompting further investigations. There was no prior history of bleeding symptoms in the patient. She was discovered to have low assays of factor V and factor VIII on subsequent investigations. Whole exome sequencing revealed the novel homozygous mutation c.604C>T in the LMAN1 gene, validating the diagnosis of F5F8D.

## Introduction

Combined factor V (FV) and factor VIII (FVIII) deficiency (F5F8D), also known as FV+FVIII, is an autosomal recessive genetic disorder characterized by a deficiency of both factor V and factor VIII. The underlying cause of F5F8D is mutations in the genes responsible for encoding two components of the endoplasmic reticulum-Golgi intermediate compartment (ERGIC-53). These genes are lectin mannose-binding protein (LMAN1) and multiple coagulation factor deficiency 2 (MCFD2) [1]. These genes form a protein complex that carries factors V and VIII from the endoplasmic reticulum to the Golgi apparatus. Consequently, the mutation impacts this transportation to the Golgi apparatus. Unlike other genetic disorders that stem from DNA defects within the genes encoding the coagulation factors themselves, F5F8D is primarily a consequence of disrupted intracellular transportation of FV and FVIII [2]. The LMAN1 gene is located on chromosome 18 (18q21) and accounts for most mutations (70%), whereas the MCFD2 gene is located on chromosome 2 (2p21) and accounts for 30% of these mutations. Diagnosis is made by measuring the levels of factors V and VIII, which typically range from 5-30% in individuals affected by this disease. A genetic study can confirm the presence of the mutated gene. Management of F5F8D is primarily symptomatic and entails the administration of fresh frozen plasma, desmopressin, and factor VIII concentrate where deemed necessary [3].

## Case presentation

We present herein a case study of a five-year-old Saudi girl referred from the otolaryngology clinic, as she was found to have prolonged prothrombin time (PT), activated partial thromboplastin time (aPTT), and international normalized ratio (INR), during routine preoperative investigations for a tonsillectomy procedure. She had no significant past medical history for any bleeding manifestations (post-vaccination hematoma, epistaxis, or any skin or muscle bleeding after trauma), bruising, or joint swelling. The physical examination was unremarkable for any bruises, mucocutaneous bleeding, or hematomas. She maintained stable vital signs and exhibited normal growth and development. Notably, her parents were in a consanguineous marriage, and her siblings did not have any history of bleeding episodes. There was no family history of any bleeding disorders. Laboratory investigations reported a normal bleeding time, a hemoglobin level of 12 g/dl, and a platelet level of 230 x 10^9/L. Coagulation profile values are outlined in Table 1.

**Table 1:** Coagulation profile PT - prothrombin time, aPTT - activated partial thromboplastin time, INR - international normalized ratio *Correction with normal pooled plasma

Coagulation profile	Result (seconds)	Reference value (seconds)	After mixing studies*
PT	18.80	11-13.5	11.70 seconds
aPTT	60.80	26-40	35.60 seconds
INR	1.5	<1.2	No result

Low levels of coagulation factors V and VIII were observed in specific coagulation factor assays, while the remaining factors exhibited normal levels, as indicated in Table 2. A whole exome sequence study was conducted that identified a novel homozygous variant c.604C>T in the LMAN1 gene, confirming the diagnosis of autosomal recessive congenital factor V and factor VIII deficiency (Table 3).

**Table 2:** Result of coagulation factor assays vWF:Ac = von Willebrand factor activity, vWF:Ag - von Willebrand factor antigen

Coagulation factors	Result	Normal range	Units
Fibrinogen	314	160-340	mg/dl
Factor II	112	70-109/	%
Factor V	<10	70-140	%
Factor VIII	14	50-150	%
Factor IX	61	50-150	%
Factor X	89	53-120	%
vWF:Ac	54	48-180	%
vWF:Ag	73	44-145	%

**Table 3:** Whole exome sequencing study MIM - Mendelian Inheritance in Man, AR - autosomal recessive, MAF - multiple allele frequency, gnomAD - Genome Aggregation Database

Gene (isoform)	Phenotype MIM number (mode of inheritance)	Variant	zygosity	MAF gnomAD	classification
LMAN1 (NM_005570.4)	227300 (AR)	c.604C>T p.(Arg202*) chr18:57020469	homozygous	0	pathogenic

Prior to the surgical procedure, the patient was administered antifibrinolytics and FVIII concentrate, followed by close monitoring in the postoperative period. No complications were observed during the postoperative phase. The patient and her family received counseling regarding her diagnosis and were recommended to attend regular follow-up appointments to monitor her factor levels consistently. Additionally, she was provided with antifibrinolytics to be utilized in the event of mucocutaneous bleeding. The family declined to undergo whole exome sequencing (WES) testing for them.

## Discussion

Combined factor V and factor VIII deficiency (F5F8D) alone are distinct from the combined deficiency of both factors, not only in terms of how they are inherited but also in the specific genes involved [1]. Factor V deficiency leads to parahemophilia, while factor VIII deficiency causes hemophilia A. F5D8D typically presents with mild to moderate bleeding symptoms and does not exhibit any other obvious clinical symptoms. This condition can manifest at any age. Interestingly, bleeding due to F5F8D is generally less severe compared to cases where factor V or factor VIII deficiency occurs in isolation. Notably, there is no observed correlation between the severity of the disease and the levels of factor V and factor VIII [2]. In most cases, patients are unaware of their condition until they experience trauma, undergo labor, tooth extraction, post-circumcision, or post-surgery, which can result in uncontrolled bleeding. It is during these instances that vigilant physicians can identify and diagnose the condition based on the presence of excessive bleeding. Spontaneous bleeding manifestations may include epistaxis, menorrhagia, easy bruising, and gum bleeding [2-4].

Laboratory abnormalities associated with this condition include prolonged PT, aPTT, and INR. However, when mixing studies are conducted, the values of PT and aPTT are corrected. Interestingly, in many reported cases, the decrease in aPTT is greater than that of PT [2]. Platelet levels and bleeding time, however, remain within the normal range [5]. FV and FVIII levels are typically decreased, usually ranging from 5 to 30% of normal. The confirmation of the diagnosis is achieved through a comprehensive whole exome sequencing study, which identifies the mutated gene as either LMAN1 or MCFD2 [6].

The prevalence of F5F8D is estimated to be 1 in 1,000,000 in the general population. While cases have been reported worldwide, the disorder is more commonly found in the Mediterranean and Middle Eastern regions. Due to its autosomal recessive mode of inheritance, parents are obligate heterozygotes, leading to an increased prevalence in areas with high rates of consanguinity [7]. F5F8D, the most prevalent type of disorder in the single-gene defects category of familial multiple coagulation factor deficiency (FMCFD) disorder, affects both males and females equally. FMCFD is a group of rare inherited bleeding disorders characterized by a concordant decrease in two or more coagulation factor levels [8]. Combined deficiency of vitamin K-dependent clotting factors (VKCFD) is also caused by single gene defects.

Treatment is tailored based on symptom severity, with prophylaxis rarely necessary. In cases where bleeding control is required, fresh frozen plasma (FFP) can be administered to replace FV and FVIII concentrate, as recommended by the United Kingdom Hemophilia Centre Doctors’ Organization [2]. A Chinese study suggests that desmopressin can effectively increase FVIII levels without the need for FFP, and it has been successfully used in patients with this condition [9]. Desmopressin can be administered before minor surgeries, labor, tooth extraction, and circumcision in patients with FVIII levels above 15% [3-5]. It is preferable to replace FVIII concentrates with recombinant or plasma-derived concentrates. Prophylaxis may be considered in cases of recurrent hemarthrosis or intramuscular hemorrhage [6]. Antifibrinolytic drugs can be used to control menorrhagia.

F5F8D may be mistakenly diagnosed as congenital factor V deficiency because of its subtle manifestations, deficient FV levels, prolonged aPTT and PT, and autosomal recessive pattern of inheritance. The situation becomes even more perplexing when FV deficiency is linked with type 1 von Willebrand disease, resulting in a concordant reduction of FVIII and FV. In such instances, genetic testing proves to be invaluable for confirming an accurate diagnosis [6].

## Conclusions

The subtle nature of this disease poses a risk of underdiagnosis, while its resemblance to FV deficiency in von Willebrand disease may lead to misdiagnosis. The primary objective of this report is to alert physicians to consider the possibility of rare diseases when assessing patients with prolonged coagulation profiles, particularly if the epidemiological data aligns with the patient's characteristics and there is a history of consanguinity among their parents. It is imperative to conduct further investigations to evaluate the efficacy of different therapeutic approaches and establish comprehensive protocols that can enhance the quality and effectiveness of patient care. Patients are strongly encouraged to comply with routine follow-ups to prevent any potential complications in the future.
